# Mollusc N-glycosylation: Structures, Functions and Perspectives

**DOI:** 10.3390/biom11121820

**Published:** 2021-12-03

**Authors:** Erika Staudacher

**Affiliations:** Department of Chemistry, University of Natural Resources and Life Sciences, 1190 Vienna, Austria; erika.staudacher@boku.ac.at; Tel.: +43-1-47654-77263

**Keywords:** N-glycosylation, mollusc, glycan structure, snail glycosylation

## Abstract

Molluscs display a sophisticated N-glycan pattern on their proteins, which is, in terms of involved structural features, even more diverse than that of vertebrates. This review summarises the current knowledge of mollusc N-glycan structures, with a focus on the functional aspects of the corresponding glycoproteins. Furthermore, the potential of mollusc-derived biomolecules for medical applications is addressed, emphasising the importance of mollusc research.

## 1. Introduction

Mollusca is a large and evolutionary very successful phylum of animals comprising about 200,000 recognised species (alive or fossil) with very heterogenous morphology (gastropods, cephalopods, bivalvia). For more than 500 million years, since the Upper Cambrian, this phylum has populated freshwater, marine and terrestrial habitats worldwide. Some species are recognized as important members of several ecosystems in terms of waste disposal and cleaning, others are used due to their nutritional value and shells [1]. One class of molluscs, the cephalopoda (squid, octopus), is used in the medical context as a model for neuronal studies. However, other species, especially snails and slugs, are ill-reputed and known as pests in agriculture or hosts of parasite life cycles.

In general, their success in survival, their adaptability to changing environmental conditions and their immense potential in medical and pharmaceutical application (see below), make molluscs an interesting target for research. However, despite their significance, there is a general lack of knowledge regarding mollusc biochemistry or molecular biology. In addition, only few genomic resources are available, further hampering research [2]. However, the investigation of mollusc biosynthetic pathways, recognition processes and interactions in general can provide valuable knowledge.

Glycosylation plays an important role in altering the properties of proteins and lipids, modifying their interactions by changing the biophysical characteristics of a target molecule significantly. Attached glycans contribute not only to physical properties, such as conformational stability, protease resistance, charge, or hydrophilicity, but also modulate several types of recognition processes ranging from reproductive biology, self/non-self-recognition, cell-cell communication, cell trafficking, development, differentiation, host-pathogen or host-symbiont interaction, immune activation, cell death and even to evolution [3,4].

N-glycosylation, one of the most common types of glycosylation, is conserved between eukaryotes. The process starts in the endoplasmic reticulum (ER) by forming a lipid-linked precursor which is transferred en bloc onto the amide of an asparagine side chain located in the consensus sequence Asn-X-Ser/Thr, where X may be any amino acid except proline. Most commonly, except for unicellular species, the precursor is a Glc_3_Man_9_GlcNAc_2_ [5]. Subsequent modifications of N-glycans are then conducted in different areas of the Golgi apparatus. In a stepwise process, specific glycosidases and glycosyltransferases sequentially add and remove monosaccharides to form high mannosidic, paucimannosidic, hybrid or complex type N-glycans [6]. Further modifications, such as methyl groups, sulphate, phosphate, phosphorylcholine or phosphoethanolamine may also occur [7,8]. The final structure of the glycan depends on the organism, the tissue, the developmental and physiological stage of the cell and even the glycosylation site within the protein. Molluscs display a very broad spectrum of such N-glycans on their proteome. They combine structural features from mammals, plants, insects and nematodes, supplementing them with additional rare features.

This publication gives an overview on the still scattered knowledge of mollusc N-glycosylation. The data are arranged around probable functions of the corresponding glycoproteins. Within the chapters, the development of the methodical approach in glycan identification can also be observed. At the beginning of carbohydrate analysis, simple monosaccharide component analysis and selected lectin binding studies were state of the art, which was then followed by the determination of detailed structures by NMR. Recently, sensitivity of the methodical portfolio has increased dramatically with wide availability of different mass spectrometry and array techniques. However, these technically sophisticated methods must be supported by further tools, for example digestion by specific exoglycosidases or chemical treatment, to confirm linkages or to distinguish isoforms [9].

Besides the focus on N-glycosylation, this review also wants to emphasise the importance of further investigations on molluscs. Hence, it is supplemented by two further short chapters on lectins and cone glycopeptides, which do not deal directly with N-glycans, but aim to highlight the importance of gastropod research. The past months of the pandemic clearly showed the need to know much more about any biological interaction, even when a species is not so important at first glance. For molluscs, we still do not know what surprises, be they treasures or hazards, they contain.

## 2. Shell Matrix Protein Glycosylation

Mollusc shells are built of calcium carbonate and an elaborated organic matrix. Shell matrix proteins, components of this organic matrix, control the necessary biomineralisation process [10]. Posttranslational modifications of these proteins, including glycosylation, often contribute to this process by modulating the protein activity [11]. The significance of glycoproteins in the biomineralisation process could be confirmed clearly by deglycosylation experiments or the comparison of glycosylated with recombinant non-glycosylated versions [12,13,14]. Because most conducted studies on shell matrix protein glycosylation present only monosaccharide compositions, ratios of sugar to protein, or lectin binding experiments, structural details and linkage information are still rare. Only two molluscs have been studied in greater detail for their shell protein N-glycosylation: the tropical freshwater snail *Biomphalaria glabrata* and the marine bivalve *Mytilus edulis*.

**Figure 1:**
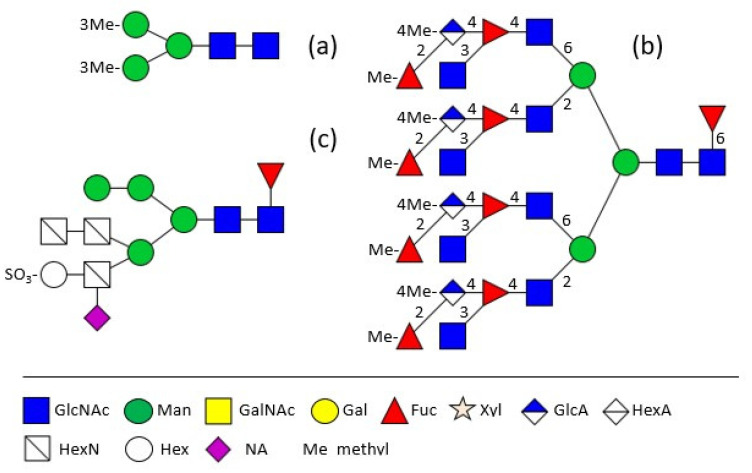
Shell matrix protein N-glycans of (**a**) *Biomphalaria glabrata* [15]; (**b**) *Mytilus* *edulis* [16]; (**c**) *Pincatada fucata* [17]. The structure plots were generated in the notation of the Consortium for Functional Glycomics (http://www.functionalglycomics.org, accessed on 16 November 2021). All figures were drawn especially for this review, based on previously published data as cited.

The major shell matrix protein of *Biomphalaria glabrata*, dermatopontin, is a 148 amino acid protein with one N-glycosylation site which is occupied by a 3-O-methylated pentasaccharide (Figure 1a) [15]. Completely different to this, the 28 kDa-sized major extrapallial protein of *Mytilus edulis* displays on its single N-glycosylation site a variation of large tetra- and penta-antennary 4-O-methylated structures with the rarely occurring structural feature of branched fucose residues (Figure 1b,c) [16]. In another marine bivalve, the Akoya pearl oyster *Pincatada fucata*, acidic compounds, namely sulphite linked to a terminal hexose as well as sialic acid, linked to a non-terminal hexosamine in the α1,3-antenna, have been identified [17]. 

## 3. Hemolymph Proteins

Hemocyanins are blue copper-containing respiratory proteins in the hemolymph of arthropods and molluscs. They are usually of high molecular weight and display a complex quaternary structure [18,19]. Due to their immunostimulating properties, which are at least partly caused by their glycans, hemocyanins are by far the best investigated proteins of snails.

Over the last decades, detailed investigation of these glycan structures has been made possible by the development and improvement of a number of analytical tools and instruments, especially mass spectrometry. Today, specific glycosylation sites within a polypeptide chain as well as minimal amounts of material can be analysed. By using these tools, mollusc glycans never cease to surprise researchers due to their various glycan structures and additional features. Here, a short overview is given, focusing in particular on new and uncommon structural features found for the first time in mollusc hemocyanins.

**Figure 2:**
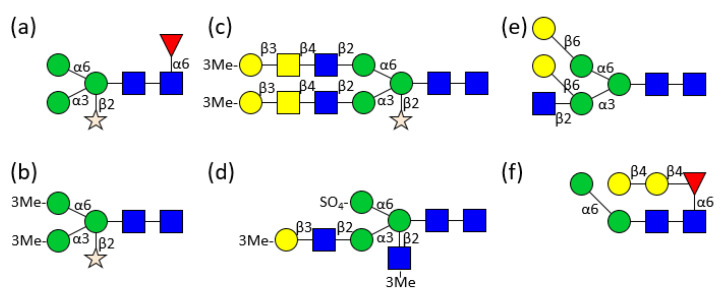
Examples for hemocyanin N-glycan structures from different molluscs. (**a**) *Helix pomatia* [21]; (**b**) *Lymnaea stagnalis* [22]; (**c**) *Lymnaea stagnalis* [23]; (**d**) *Rapana venosa* [27]; (**e**) *Megathura crenulata* [28]; (**f**) *Megathura crenulata* [29]. For the symbols key see Figure 1.

Detailed analysis started at the end of the seventies with the identification of 3-O-methylated mannose and 3-O-methylated galactose in the course of monosaccharide analyses of *Helix pomatia* and *Lymnaea stagnalis* glycans [20]. Then, in the mid-eighties, the first glycan structure was determined by 500 MHz 1H NMR spectroscopy. Xylose, known from cartilage and plants, was found in the *Helix pomatia* α-hemocyanin (Figure 2a) [21], followed by the identification and exact location of 3-O-methylated mannoses in the hemocyanin of the freshwater snail *Lymnaea stagnalis* (Figure 2b) [22]. 3-O-methylated galactose could be found in *Lymnaea stagnalis* as well (Figure 2c), but also in the marine snail *Rapana thomasiana* (today named *Rapana venosa*) and to an even higher extend in the land snail *Helix pomatia* [23,24,25]. In *Helix pomatia* 4-O-methylated galactose was determined, additionally [25]. Another feature, 6-O-methylated mannose, which is a constituent of the otherwise high mannosidic glycans of tridacnin, a lectin found in the hemolymph of the clam *Hippopus hippopus*, could be identified as well [26]. At first they were considered a novelty, but by now xylose and methylated hexoses have been found in many other molluscs and are recognised as a typical feature of mollusc glycans. 

Even more unusual structures were found in the functional unit of RvH_1_-a of *Rapana venosa* hemocyanin. Besides a biantennary structure terminated by two methylated galactoses, a sulphated glycan with a methylated bisecting GlcNAc was identified (Figure 2d) [27].

Analysis of keyhole limpet (*Megathura crenulata*) hemocyanin revealed another two novel modifications. Firstly, a galactose residue directly β1,6 linked to mannose was identified on high mannosidic or hybrid N-glycans, some substituted by an additional core α-fucosylation (Figure 2e) [28] and, secondly, a chain of two β1,4 linked galactoses substituting the α1,6 linked core fucose (Galβ1,4Galβ1,4Fucα1,6-) of another group of structures, was found (Figure 2f) [29]. A single galactose linked to the inner fucose had already been seen before in another protein derived from mollusc origin, the octopus’s rhodopsin, but this structure with two galactoses had been completely unknown until then [30].

**Figure 3:**
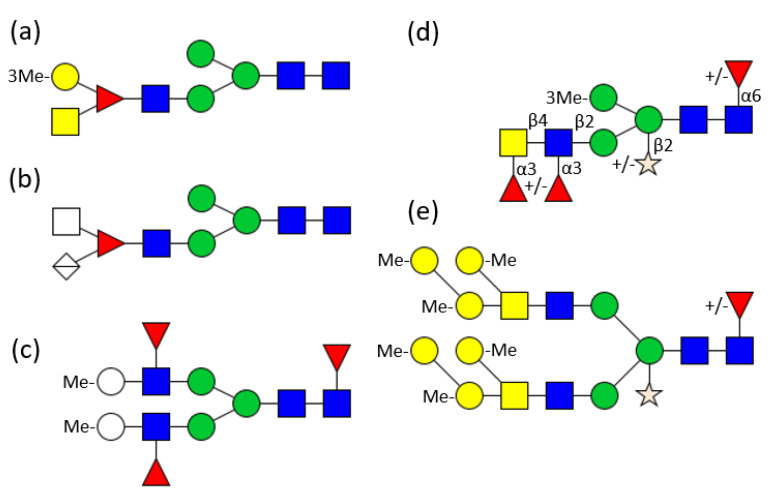
Examples for hemocyanin N-glycan structures from different molluscs. (**a**) 7 RtH2-e of *Rapana thomasiana* [31]; (**b**) RvH_1_ and RvH_2_ of *Rapana venosa* [32,33], (**c**) HtH1 of *Haliotis tuberculata* [34], (**d**) *Biomphalaria glabrata* [35], (**e**) *Helix pomatia* [25,36]. For the symbols key see Figure 1.

Soon afterwards, not only mono-substituted, but also branched fucoses were identified in the glycan antennae. RtH2-e, the functional unit of *Rapana venosa* hemocyanin, contains a hybrid glycan structure with a fucose located in the α1,3 antenna substituted by GlcNAc as well as by 3-O-methyl-galactose (Figure 3a) [31]. In RvH_1_ and RvH_2_, however, the fucose is substituted by an amino sugar and a hexuronic acid (Figure 3b) [32,33]. In contrast to that, HtH1, the hemocyanin functional unit of *Haliotis tuberculata*, displays the more common structure with unsubstituted fucoses linked to the antennal GlcNAc residues (Figure 3c) [34].

*Biomphalaria glabrata* hemolymph hybrid N-glycans mostly carry a xylose in the 2-position to the central mannose and an α1,6 fucose linked to the innermost GlcNAc of the core. Their α1,3 linked antenna is elongated by a GalNAc-GlcNAc moiety, with both amino sugars fucosylated (Figure 3d) [35], whereas *Helix pomatia* displays up to six methylated galactoses on the antennae (Figure 3e) [25,36]. Similar structures decorate the 13 potential glycosylation sites of the β HlH subunit of *Helix lucorum* hemocyanin. The inner core of the 32 different identified glycans is often α1,6 fucosylated and/or β1,2 xylosylated. A significant number of these glycans is of the hybrid type with only one mannose on the α1,6 antenna, but a GalNAc-GlcNAc elongation, terminated by several methylated hexoses, on the α1,3 antenna [37].

A recent review compares on a very high technical level N-glycan structures, glycan-protein binding sites and conformational aspects of hemocyanins found in the Black Sea snail *Rapana venosa*, the garden snail *Helix lucorum* and the abalone *Haliotis tuberculata* [38]. All investigated organisms show a high content of methylated hexoses (3-O-methyl mannose and 3-O-methyl galactose) and acidic structures. In fact, comparative analysis of amino acid sequences, close to the putative N-glycosylation sites, on the surface of the functional units show a high degree of homology, thus implying an important contribution of the glycans to the function [38].

Hemocyanins carrying typical mollusc structural features on their glycans, such as fucosylation, xylosylation or methylated hexose residues, display strong immune stimulatory properties in animal studies and therefore, are used to enhance the efficiency of anti-tumour vaccines [39]. The importance of glycans and their contribution to the structure of these large protein complexes became evident in deglycosylation experiments. Deglycosylation of hemocyanins from different mollusc origin (*Rapana thomasiana*, *Concholepas concholepas*, *Fissurella latimarginata*, *Megathura crenulata*) disrupts the quaternary structure of the protein complexes and reduces their immunogenic capacities. Especially methylated galactoses and xylose seem to be the key players of this effect [36,40,41]. In vitro studies on hemocyanins from the marine snail *Rapana venosa* and the garden snails *Helix aspersa* and *Helix lucorum* revealed antitumor effects on human colorectal adenocarcinoma cells [42]. A recent study shows that hemocyanins from *Concholepas concholepas* and *Fissurella latimarginata* are safe and useful carriers of carbohydrate mimotopes and therefore new suitable candidates for the enhancement of the immunogenicity of peptides in cancer vaccine research [43]. For the last several years, keyhole limpet hemocyanin from *Megathura crenulata* has been successfully utilised for treatment of various types of cancer. These treatments have led to enhanced recognition of the importance of carbohydrates [for a review see [44]. In the specific case of superficial bladder cancer, however, the involvement of glycans on the hemocyanins from *Megathura crenulata*, *Concholepas concholepas* and *Fissurella latimarginata* seem not be necessary to bias the immune response towards the cancer cells through natural, complement-activating antibodies [45].

Research on immuno-stimulative properties of mollusc glycoproteins is ongoing and expanded to additional applications. Besides cancer treatment, the immune stimulatory benefits of snail hemocyanins have been successfully evaluated for viral infections. *Rapana venosa* RvH2-e, for example, which contains glycans essential for its activity, exhibits an antiviral effect against herpes simplex virus type 1 (HSV-1) [33].

## 4. Glycosylation of Other Mollusc Proteins

Much less is known about N-glycosylation of other proteins. Multiple studies compared N-glycan patterns of complete adult tissue preparations (viscera and skin) of terrestrial slugs (*Arion lusitanicus*, *Limax maximus*), terrestrial snails with shells (*Cepaea hortensis*, *Arianta arbustorum*, *Achatina fulica*) and a freshwater snail (*Planorbarius corneus*) for their similarities and differences. All analysed snails contained mainly high mannosidic and paucimannosidic structures terminated by 3-O-methylated mannose residues. The inner core was decorated with a β1,2-linked xylose at the β-mannose and often fucosylated at the inner GlcNAc, mainly in α1,6 position. Only in *Limax maximus* and *Achatina fulica* some larger structures containing galactose and/or 3-O-methylated galactose were observed [46,47]. Neither in *Arion lusitanicus* eggs nor on the extracellular coat of the bivalve *Unio elongatulus* were methylated hexoses discovered, thus glycosylation patterns undoubtedly are developmentally regulated and vary between different stages [46,48]. This finding is supported by a study on *Achatina fulica* eggs, young snails and adults showing remarkable differences in their glycosylation patterns [49].

Rhodopsin, the visual pigment in photoreceptor cells of the cephalopod *Todarodes pacificus*, has been studied as well and is mainly glycosylated (85%) by a α1,3/α1,6 difucosylated pentasaccharide core with the α1,6 fucose elongated by a galactose residue in β1,4 position. No methylated sugars were detected here [50].

**Figure 4:**
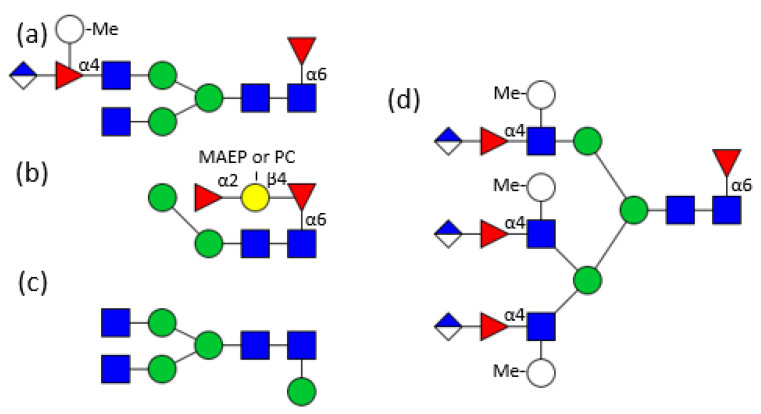
(**a**–**d**) Examples of *Volvarina rubella* glycans [51]; ]. For the symbols key see Figure 1; MAEP, Methylaminoethylphosphonate; PC, phosphorylcholine.

A whole glycome analysis was performed on the total tissue of the marine bivalve *Volvarina rubella* revealing a high complexity of about 100 different N-glycan structures. While the common mollusc structural characteristics, such as xylose or methylation of hexoses were either not present or in low abundance respectively, some new features were identified. Additional mannose residues were found linked to the innermost GlcNAc in α1,3 position and peripheral in an unusual α1,6 position. The α1,6 linked fucose of the inner core was not only substituted by a β1,4 linked galactose, but the chain was further substituted by an α1,2 linked fucose residue. Phosphorylcholine and up to four *N*-methyl-2-aminoethylphosphonate molecules on mannose and GlcNAc residues increased the heterogeneity. Additionally, sulphatation of mannose residues or trisaccharide units containing a branched fucose substituted by a methylated hexose and uronic acid were revealed to be responsible for the charge of anionic N-glycans (Figure 4a–d) [51]. 

So far, nothing is known about the function of these unusual decorations of N-glycans found in the different studied species. Presumably, similar to all other phyla, glycans in molluscs play an important role in many recognition processes within and among cells. It is clear, however, that at least neuronal processes are influenced by glycoproteins in snails. *Helix pomatia* nervous tissue contains an N-glycosylated glycoprotein (F3/contactin immunologically related protein, HCRP), which is involved in axonal growth control and neurotransmitter release and in *Lymnaea stagnalis* an increase of glycosylation during long–time-memory formation was detected. However, no detailed information is given regarding the glycan structures involved [52,53].

## 5. Molluscs as Intermediate Hosts for Parasites

Snails are the intermediate hosts for several parasites, mainly trematodes, which need the molluscs for some steps of their life cycle. In many of the diverse recognition events during this process, for example, recognition of a proper host, adhesion or invasion, protein linked carbohydrates are at least one of the binding partners.

*Biomphalaria glabrata* is the intermediate host of the trematode *Schistosoma mansoni*, the causative agent of human schistosomiasis, which affects hundreds of millions of people worldwide. Hence, it is the best investigated intermediate host/parasite system. The snails are essential for asexual reproduction in this parasite’s life cycle. [54,55,56]. Because of its human relevance, a *Biomphalaria glabrata* embryonic (Bge) cell line, the only established cell line from mollusc origin, is available for research [57].

Several galectins, lectins or lectin-like proteins have been found in snails which play a role in the recognition of parasite-specific glycan motifs and may also be correlated to susceptibility or resistance of snail strains towards infection by the parasite [58]. Several glycan structures have been identified which play major roles in the blood fluke—snail interaction [59,60,61,62]. For example, a tandem-repeat like galectin was identified in Bge cells, as well as in circulating hemocytes of adults of *Biomphalaria glabrata*, displaying Lac or Gal binding activity [63]. These N-glycans of hemolymph glycoproteins, which cross react with *Schistosoma mansoni* glycoconjugates, are paucimannosidic or hybrid structures containing often an α1,6 linked fucose and/or a β1,2 xylose linked to the core pentasaccharide. The α1,3 antenna of the glycan is elongated by a LacdiNAc unit (GalNAcβ1,4GlcNAcβ1-) containing up to two α1,3 linked fucose residues (Figure 3d) [35]. These structural features are shared with *Schistosoma mansoni* sporocytes and miracidia, two snail-pathogenic larval stages of the parasite [64]. Further studies demonstrated that especially the fucose residues are essential for the interaction between host and parasite [65]. Enhanced occurrence of glycans with two or three fucose residues on the hemolymph glycoproteins of the Puerto Rico *Biomphalaria glabrata* strain (BgPR) are correlated with the high susceptibility of this strain towards parasite infection [66].

The great pond snail *Lymnaea stagnalis* is the intermediate host for the larvae of a variety of bird schistosomes (genus *Trichobilharzia*), the causative agent of cercarial dermatitis, as well as for the liver fluke *Fasciola hepatica*, a common parasite in ruminants and also humans [67,68]. As a reaction to infection, *Lymnaea stagnalis* down-regulates cell signalling, which is at least partly caused by fucose and galactose [69]. *Fasciola hepatica* and its further host, *Galba truncatula*, display structural similarities of surface carbohydrate residues in the contact zone between both organisms. This suggests that carbohydrate mimicry is used by the parasite to evade host immune response [70].

**Figure 5:**
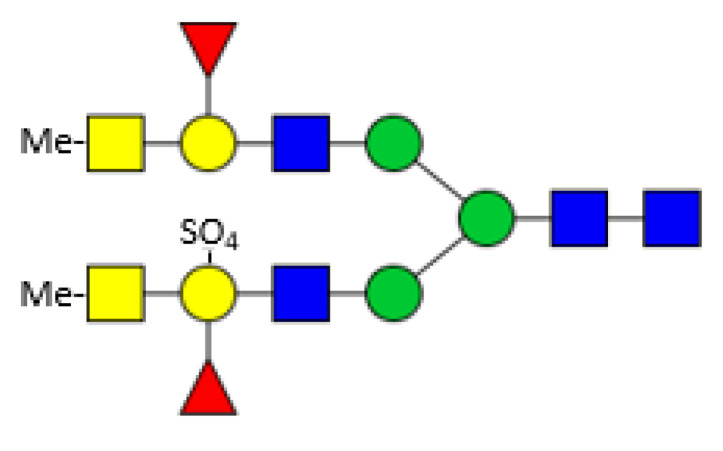
Example of a *Crassostrea virginica* glycan [74]; For the symbols key see Figure 1.

Eastern oysters (*Crassostrea virginica*) contain two galectins (CvGal1 and CvGal2) which are involved in the immunological response upon infection by the protozoan parasite *Perkinsus marinus*. CvGal1 binds in vitro strongly to type-2 blood group A oligosaccharides, but in the biological context binding to type-1 blood group A glycans may be preferred [71,72]. CvGal2 displays a broader specificity and can distinguish between different *Perkinsus* species [73]. The oyster plasma and hemocyte glycans comprise a broad spectrum of paucimannosicic, hybrid and complex structures containing core α1,6 fucosylation, core α1,3/α1,6 difucosylation and some structures with an α1,3 linked hexose at the innermost GlcNAc residue. The antennae are often terminated by methylated GalNAc residues. The acidic glycans are substituted by one or more sulphate groups linked to terminal or innermost galactose residues comprising modified forms of human histo-blood group A antigens (Figure 5) [74]. These glycans fit perfectly to the specificity of the previously mentioned *Biomphalaria glabrata* galectins. Surprisingly, no xylose could be detected here.

In addition, different nematodes use snails, for example *Achatina fulica*, as intermediate hosts in their life cycles, but no detailed information on glycan involvement is given [75]. 

Keyhole limpet (*Megathura crenulata*) is not susceptible to *Schistosoma mansoni* infection, but its hemocyanin glycans cross-react serologically with glycoconjugates of this parasite. The epitope responsible for this was identified as the Fucα1,3GalNAcβ1,4(Fucα1,3)GlcNAcβ1- motif. Using the cross-reaction behaviour, keyhole limpet is a valuable tool in diagnosis and may act as a model antigen for further studies on immunopathological mechanisms [76]. Our knowledge about intermediate host-pathogen interaction processes is still scattered. Efforts in this research area need to be increased in order to provide ways to interrupt parasite life cycles, to avoid tremendous numbers of infections worldwide. 

## 6. Enzymes Involved in N-glycan Biosynthesis

By now, the decreasing costs of DNA sequencing has enabled full sequencing of various species. However, for molluscs there is still much less genetic information available, compared to other phyla. Fewer than 50 studies describe partial or complete genome mollusc assemblies, for example those of the Pacific oyster *Crassostrea gigas* (synonym *Magallana gigas*) or the freshwater snail *Biomphalaria glabrata*, the intermediate host of the human blood fluke *Schistosoma mansoni* [2,77,78]. From the latter source an immortalised cell line has been developed (*Biomphalaria glabrata* embryonic cell line—Bge), which thus is the only available lophotrochozoan in vitro cell model. However, it must be emphasised that the laboratory strain differs both in sequence and structure from the reference genome [79]. For many other mollusc species only limited data are available so far. The missing opportunity for homology search is one main reason why the knowledge of enzymes and their characteristics is so limited within this phylum.

Molluscs are important players as waste removers in the environment. To accomplish this, they contain a number of oligosaccharide degradation enzymes, such as mannanases, cellulases, chitinases or glucosidases. Several such exo- and endoglycosidases have been identified, characterised and partly recombinantly produced. As this review mainly focuses on N-glycosylation, here only enzymes taking part in the N-glycosylation process are described.

In respect of N-glycan biosynthetic pathway enzymes, only the freshwater snail *Lymnaea stagnalis* has been well investigated. Several enzymes responsible for the formation of typical complex mollusc glycans were identified in the connective tissue of this snail. In nearly all phyla, including molluscs, biosynthesis of complex N-glycans is initiated by the transfer of a GlcNAc residue to the α1,3 antenna of an N-glycan as prerequisite for further enzymes. GlcNAc-transferase I (β1,2-*N*-acetylglucosaminyltransferase I) action then allows subsequent modification by GlcNAc-transferase II (β1,2-*N*-acetylglucosaminyltransferase II) and β1,2-xylosyltransferase [80]. Terminal GlcNAc residues can be further elongated by β1,4-GalNAc-transferase (UDP-GalNAc: GlcNAc β-R β1,4-*N*-acetylgalactosaminyltransferase) forming a LacdiNAc-chain (GalNAcβ1,4GlcNAc), which occurs frequently in invertebrates [81,82]. Onward elongation is performed by β1,3-galactosyltransferase [83]. In addition, three types of fucosyltransferases have been described. Two of them act on terminal Gal or GlcNAc residues (GDP-Fuc:Galβ1,3GalNAc-R(Fuc to Gal) α1,2 fucosyltransferase and GDP-Fuc:Gal β1,4GlcNAc (Fuc to GlcNAc) α1,3 fucosyltransferase [84], while the third one (core α1,3 fucosyltransferase) adds a fucose onto the innermost GlcNAc residue forming an α1,3 core fucosylation [85]. Even though many glycans have been identified, which contain fucose α1,6 linked to the inner GlcNAc, the corresponding α1,6 fucosyltransferase in mollusc species is still unknown.

In contrast to vertebrates, where GlcNAc residues of the antennae are usually substituted by Gal residues, in molluscs a terminal GalNAc residue is frequently added. Interestingly, the responsible enzyme, the β1,4-GalNAc-transferase, shows high DNA homology and structural similarity to the mammalian β1,4-galactosyltransferase [81,82]. In addition, a β1,4-GlcNAc-transferase with clear homology to the mammalian β1,4-galactosyltransferase was identified, but this enzyme is more likely involved in O-glycan biosynthesis [86]. In the prostate gland of *Lymnaea stagnalis* a β1,4-glucosyltransferase was found, which can glucosylate terminal GlcNAc residues of O-glycans as well as of N-glycans. However, the products of this enzyme’s activity could not be determined in tissues so far [87].

An essential requirement requisite for the production of any glycan is the availability of the appropriate activated sugar nucleotides. One of these vital genes was identified in the genome of the Pacific oyster *Magallana gigas* (synonym: *Crassostrea gigas*). It was proven to encode UDP-Glucose-4-epimerase as the corresponding protein could catalyse the conversion of UDP-Glc into UDP-Gal [88]. In the same study, another gene, a β1,4-galactosyltransferase from oyster, was expressed and purified but not checked for activity on N-glycan substrates [88].

Overall, the biosynthesis of N-glycans in molluscs seems to obey in general what we know from other phyla regarding the first basic steps. When the N-glycosylation process of mammals was compared to that of *Mytilus galloprovincialis* digestive cells along the Golgi apparatus, only a handful of differences were found: a lack of sialic acid, a very prompt galactosylation and an increased fucosylation of the antennae [89]. However, even though the first steps are quite similar, further modifications of the glycans are far more variable in terms of compounds (sugars and other additions, such as methyl or sulphate groups) and possible linkages. The consequence is a much wider spectrum of possible glycan structures than identified in vertebrates or plants.

Currently the only application for mollusc enzymes discussed is a usage of the impressive transglycosylation ability of some glycosidases. This would enable an easy and environmentally friendly production of various disaccharides. For example, the β-D-glycosidase from the China white jade snail (a special breeding of *Achatina fulica*) can transfer fucose, glucose as well as galactose to different acceptor substrates [90].

Due to the broad spectrum of glycan structures in molluscs, it can be assumed that a number of glycosyltransferases with new specificities are present. Those enzymes, recombinantly expressed, would allow new advances in chemoenzymatic synthesis of glycans which may be useful for diagnosis and treatment of several diseases.

## 7. Current and Prospective Applications of Mollusc Lectins

Lectins are proteins that bind specifically to carbohydrate structures. They are part of numerous recognition and adhesion processes in all phyla. Their strict restriction to specific sugars in specific linkages allows a high selectivity and discrimination for interactions in the living organism, but also provide a powerful tool for basic research, diagnostic and therapeutic applications. Already for more than forty years, lectins from mollusc origin have been isolated and characterised. Presumably, they play a crucial role in innate immune protection. Due to their binding specificity, they are targeted to structural elements characteristic for bacteria. For this purpose, sialic acid or galactose-binding lectins are found in many mollusc species (*Achatina fulica*, *Cepeaea hortensis*, *Limax flavus*, *Mytilus californianus*) with a putative role in the defence against microorganisms [91,92,93,94,95]. An upregulation of a sialic acid-binding lectin of *Crassostrea hongkongensis* is visible upon bacterial infection [96]. In *Achatina fulica* a high molecular weight lectin specific for galactose does not inhibit bacterial growth, but rather induces agglutination of the microorganisms [97]. Furthermore, lectins also have other tasks in molluscs, for example the protection of eggs by an anti-nutritive defence function. A highly stable, non-digestible lectin was described and characterised revealing this function in *Pomacea diffusa* [98]. For a review on carbohydrate recognition in mollusc immune defence see [99].

Many of these lectins have the potential to be used as powerful tools in medicine. Malignant transformations of cells often correlate with an altered glycosylation pattern on the surface of those cells. Lectins, which specifically bind to these structures, meanwhile are used in diagnosis and treatment of various kinds of cancer. This includes several mollusc derived lectins which are under investigation or already in application as biomarkers. For example, a *Helix pomatia* lectin, which recognises O-GlcNAc residues in metastatic breast cancer and a *Helix aspersa* lectin, which recognises O-GalNAc on various cancer cells are already used for diagnosis [100,101,102,103,104].

A completely different potential application, but nonetheless important, is possible with the C-type hemolytic lectin from the freshwater clam *Villorita cyprinoides*. This lectin has a significant clot lysis activity and could be developed as a new antithrombotic drug molecule [105].

## 8. Glycopeptides from *Conus* Marine Molluscs

Predatory cone snails (genus: *Conus*) use complex venoms to paralyse and kill their prey. These venoms are mostly composed of multiple small peptides with a high content of disulphide bridges and posttranslational modifications. Due to the high diversity between Conus species, the total number of different existing peptides was estimated to be around 50,000 [106]. Many of the already identified peptides are mucin-type O-glycosylated, but no N-glycosylation has been described so far [107,108]. Some of the peptides interact with nicotinic acetylcholine receptors or are able to desensitise neurotensin receptors [109,110]. Peptides with low toxicity but a capacity to block these receptors irreversibly may be of pharmaceutical use in a broad field of applications, such as pain, epilepsy, and myocardial infarction. For a detailed review on cone toxins see [111].

## 9. Conclusions

Molluscs are able to produce all kinds of N-glycan structures ranging from those typical in mammals to structures similar to those found in plants, insects or nematodes. They also add further interesting features to the portfolio of glycosylation in living cells. This makes them a valuable model for research on N-glycosylation in general and may help to understand several interaction processes between cells or organisms, such as fertilisation, immunomodulatory activity or host-parasite recognition events.

Potential medical applications of mollusc components range, due to the high immunomodulatory capacity, from diagnostic and therapeutic tools in cancer to models for neuroscience research (cephalopoda) or vaccines against parasites. Furthermore, antimicrobial peptides derived from snail origin may contribute significantly to the development of novel antimicrobial therapies, especially in times of increasing numbers of antibiotic-resistant bacteria and antibiotic-allergic patients.

However, other fields also could benefit from mollusc research, for example material sciences. Some interesting inputs might be gained from shell formation for the development of new robust and stable biocompatible materials or from the mucus for the development of different kinds of surface lubrication.

Last, but not least, gastropods may play an increasing role in future protein supply solutions, perhaps a necessity for the nutrition of a growing world population.

Mollusc glycosylation is a treasure chest of different structural features and combinations thereof. We are far away from understanding their function, but some of these glycoproteins might be promising candidates for future applications in human health. The detailed knowledge of this very complex glycosylation system could become a valuable tool to understand the principal rules of glycosylation in all organisms. However, the challenging and time-consuming analysis methods, the limited genetic data and the unavailability of fitting bioinformatics tools, most based only on mammalian structural features, currently hinder a fast progress in mollusc research. Nevertheless, the evolutionary, medical and veterinary importance of this phylum is worth the investment of money and time to advance research.

This review gives an overview on the current knowledge on mollusc N-glycosylation, in terms of structure, corresponding enzymes and putative function. However, we are still far away from forming a complete picture of this immense glycosylation potential. Increasing scientific effort in this complex field will be a benefit for advancing glycoscience in general.

## Abbreviations

Fuc, fucose; Gal, galactose; GalNAc, N-Acetylgalactosamine; Glc, glucose; GlcA, glucuronic acid; GlcNAc, N-Acetylglucosamine; Hex, hexose; HexA, hexuronic acid; HexN, Hexosamine, Man, mannose; Me, methyl; NA, neuraminc acid; Xyl, xylose.
